# Subsurface Life on Earth as a Key to Unlock Extraterrestrial Mysteries

**DOI:** 10.1111/1751-7915.70286

**Published:** 2025-12-18

**Authors:** Emeline Vidal, Melody R. Lindsay, James A. Bradley, Magdalena R. Osburn, S. Emil Ruff

**Affiliations:** ^1^ Marine Biological Laboratory Ecosystems Center and J Bay Paul Center for Comparative Molecular Biology and Evolution Woods Hole Massachusetts USA; ^2^ Bigelow Laboratory for Ocean Sciences East Boothbay Maine USA; ^3^ Aix Marseille Université, Université de Toulon, CNRS, IRD, MIO Marseille France; ^4^ School of Biological and Behavioural Sciences Queen Mary University of London London UK; ^5^ Department of Earth, Environmental, and Planetary Sciences Northwestern University Evanston Illinois USA

**Keywords:** astrobiology, deep biosphere, extremophiles, microbial ecology, scientific drilling

## Abstract

The first forms of life on Earth were microbial, preceding the evolution of multicellular life by more than two billion years. Based on our current understanding of the origin of life, it is likely that the first life forms on any extraterrestrial world would also be microbial. Due to the extreme temperatures, radiation or aridity on most planetary surfaces, such extraterrestrial microbes would most likely dwell in subsurface environments. Earth's subsurface features a wide range of environments, including deep marine sediments, crustal aquifers, rock fracture fluids, hydrocarbon reservoirs, caves and permafrost soils. These environments are known to host an immense diversity of life forms, predominantly microbes that survive or even thrive under extreme conditions and energy scarcity. Life's ability to endure and possibly evolve in Earth's subsurface lends credence to the possible existence of life beyond our planet and provides a blueprint for the extraterrestrial life forms and biosignatures we might expect. The exploration of space via extraterrestrial samples analysed on Earth, in situ extraterrestrial analyses, and remote sensing continue to advance our search for and understanding of potential biosignatures on other planetary bodies. But by investigating Earth's deep, dark and isolated ecosystems, we not only broaden our understanding of life's adaptability but also refine our strategies and technologies for detecting life on other planets and moons. Subsurface exploration is not just a frontier of Earth science—it is a cornerstone of astrobiology and in the pursuit of understanding the multitude of processes that could create and sustain life anywhere. In this opinion article, we discuss the latest highlights in subsurface research and technology, how Earth's subsurface environments serve as models for potential environments on other planetary bodies, why insights into subsurface microbiomes inform the search for life elsewhere, and which technologies and developments will advance the field in the future.

## Graphical abstract

This paper discusses current research on subsurface microbiology. Exploring Earth's deep and extreme subsurface environments provides critical insights into life's limits and adaptability. Subsurface exploration is not only vital for understanding the origins and limits of life on Earth, but also crucial for informing future missions in the search for biosignatures on Mars, icy moons and exoplanets. Image courtesy of the NOAA Office of Ocean Exploration and Research and of NASA.

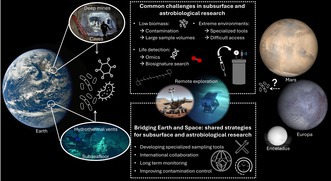

## 1 Meet the Intraterrestrials: Who Are the Microbes Living Deep Beneath Our Feet?

The subsurface microbiome is estimated to account for the majority (~85%) of Earth's global microbial biomass and may comprise up to 10^12^ species (Bar‐On et al. 2018; Magnabosco et al. 2018). Subsurface microbes play central roles in Earth's biogeochemical cycles (D'Hondt et al. 2002) and often affiliate with uncultured phylogenetic lineages (Ruff, de Hrabe Angelis, et al. 2024). Deep subsurface ecosystems harbour organisms capable of withstanding environmental extremes, including temperatures from −15°C to 122°C (Merino et al. 2019), pH values from as low as 0 (Johnson 2012; Robbins et al. 2000) to as high as 12 (Takai et al. 2001; Thieringer et al. 2023), salinity ranges from 0% to 49.7% (Merino et al. 2019), pressures from 0.1 to 340 MPa (Merino et al. 2019; Plümper et al. 2017), restriction of physical space, and high heavy‐metal concentrations (Benyehuda et al. 2003; Hemme et al. 2010). For example, *Candidatus* Desulforudis audaxviator, a sulphate reducer initially detected in 3‐km deep hypersaline waters of South African mine (Chivian et al. 2008; Moser et al. 2005), is globally widespread, yet almost exclusively occurs in subsurface environments (Karnachuk et al. 2019; Labonté et al. 2015; Lindsay et al. 2024; Ruff, de Hrabe Angelis, et al. 2024; Tiago and Veríssimo 2013; Westmeijer et al. 2024). Remarkably, *Cand*. D. audaxviator populations from distant sites are genomically very similar, though not identical, consistent with slow evolution across millions of years (Becraft et al. 2021). Deep life harbours intriguing metabolic capabilities, like the autotrophic production of acetate from hydrogen and CO_2_ (Colman et al. 2022), the production of molecular oxygen in the absence of light (Ruff, Schwab, et al. 2024), and mineral targeting metabolisms (Casar, Momper, et al. 2021). Viruses, fungi and even multicellular eukaryotes contribute to subsurface biodiversity and biogeochemical cycling (Borgonie et al. 2011; Kiel Reese et al. 2021; Labonté et al. 2015; Nigro et al. 2017; Rédou et al. 2014), despite being isolated and not in exchange with surface environments for millennia.

Microbial life in Earth's deep subsurface generally persists under conditions of profound energy and nutrient limitations. Unlike surface ecosystems powered by sunlight and rapid nutrient cycling, subsurface microorganisms subsist on vanishingly small energy fluxes, which can be as low as tens to hundreds of zeptowatts (10^−21^ W) per cell (Bradley et al. 2020; Hoehler and Jørgensen 2013; LaRowe and Amend 2015a). For comparison, an *Escherichia coli* cell in stationary‐phase in the laboratory uses ~10^−13^ W (Deng et al. 2021), which is 1–10 million times greater power. Biomass turnover, that is a cell generation time, in the deep biosphere can therefore occur on timescales of hundreds to thousands of years (Lever et al. 2015; Lomstein et al. 2012) as compared to the ~20 min in an exponential‐phase *E. coli* culture.

Cells remain alive not by growing but by carrying out essential maintenance, repair and preservation processes (Bradley et al. 2019; LaRowe and Amend 2015b). The ultra‐low‐power lifestyle of the deep biosphere challenges our conventional definition of life: growth, reproduction and active metabolism and pushes the limits of what is compatible with active life on Earth. This lifestyle establishes the deep biosphere as the largest and slowest‐living microbial biosphere on the planet.

## 2 Space Exploration and Subsurface Research Promote Collaboration, Peace and Understanding Between Nations

**FIGURE 1:**
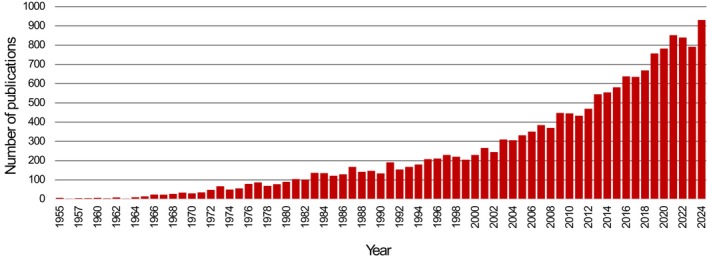
Publications using the term *subsurface* in their title over the years (values retrieved from Web of Science, June 2025).

The search for extraterrestrial life provided a major rationale for establishing the National Aeronautics and Space Administration (NASA) in 1958 which funded the first exobiology grant in 1959. Just as space exploration relied upon international cooperation, subsurface science that seeks to interrogate analogues for life on other celestial bodies greatly depends on international collaborations. Large‐scale projects including the International Ocean Discovery Programme (IODP), International Continental Scientific Drilling Programme (ICDP), the Center for Dark Energy Biosphere Investigation (C‐DEBI), the Deep Carbon Observatory (DCO), the International Census of Deep Life (CoDL) and most recently the International Center for Deep Life Investigation (IC‐DLI) have influenced decisions about where and how to seek evidence of microbial life beyond Earth (Table S1). Between 1955 and 1964, fewer than 10 publications included the term *subsurface* in their titles. Since then, the field has dramatically expanded, with more than 900 publications recorded in 2024 (Figure 1). Today, scientists recognise subsurface ecosystems as dynamic and complex environments that can offer valuable insights into extreme microbial life, biogeochemical cycles, the origin of life and the potential for life elsewhere. As our understanding deepens, more focus is given towards subsurface Earth's analogues to explore dynamics and constraints that could shape life in extraterrestrial systems. Despite these advancements and a near exponential increase in subsurface‐related investigations (Figure 1), Earth's subsurface remains the largest yet least explored biome due to its vastness and poor accessibility. Like space missions, the scientific exploration of the subsurface biosphere will benefit from sustained engineering investment and global attention. Just like space exploration, the large‐scale, international endeavours and global projects in subsurface research are promoting ‘Völkerverständigung’, peace and understanding between nations.

## 3 Subsurface Environments on Earth as Analogues for Extraterrestrial Habitats

Earth's subsurface habitats can serve as extraterrestrial analogues when their geologic and geochemical characteristics resemble those of potential extraterrestrial habitats. Analogues to potential current and past habitable areas of Mars occur across Earth's continental crust from relatively shallow depths to kilometres deep (Table 1) (Wright et al. 2024). These environments are Mars analogues due to their geological context, mirroring sedimentary and crystalline aquifers (Wynne et al. 2022). Various terrestrial settings have been explored as analogues, including the cool cratonic rocks of Archean shields—home to some of the deepest and most isolated microbial ecosystems on Earth (Lollar et al. 2019; Nisson et al. 2023). Other early studies focused on the aquifers of the Columbia River flood basalts, where subsurface lithoautotrophic microbial ecosystems were controversially identified (Stevens and McKinley 1995). Subsequently, deep life dependent on water–rock interactions was discovered in fracture fluids that have been isolated in rock of the Witwatersrand Basin, South Africa for more than one billion years (Lau et al. 2016; Lin et al. 2006; Nisson et al. 2023) (Figure 3A) and the Canadian Shield (Higgins et al. 2025; Mueller et al. 2024) as well as in deep crystalline bedrocks of the Fennoscandian Shield (Itävaara et al. 2011; Purkamo et al. 2020) and many other crustal aquifers of diverse geochemical context. Studies of obducted marine crust including the Ophiolites of Oman and the California Coast Ranges have expanded our knowledge of life within ultra‐alkaline fluids that create novel biophysical challenges for resident microbes (Colman et al. 2025).

**TABLE 1:** Extraterrestrial bodies of astrobiological interest and their potential Earth subsurface analogues.

Celestial body	Detected chemical species	Estimated temperature (°K)	Estimated depth (km)	Estimated pressure (MPa)	Estimated pH	Environmental features; geology	Potential subsurface Earth analogues	References
Europa	H_2_, O_2_, H_2_O_2_, CH_4_, NH_3_, ${\mathrm{SO}}_{4}^{2-}$, Na^+^, Cl^−^	Surface: 110 Subsurface ocean: 210–270	Ice layer: 15–25 Subsurface ocean: 100	110–260	4–6	Silicate mantle Metallic core Tidal heating	Mariana Trench (274 K, 110 MPa) Lake Vostok (270 K, 40 MPa), Mid‐Cayman Rise hydrothermal field (25–50 MPa)	Marion et al. (2003, 2005), Carr et al. (1998), Carlson et al. (1999), Weber et al. (2023), Barge and White (2017), Nimmo et al. (2003) and Anderson et al. (1998)
Enceladus	H_2_O, CH_4_, CO_2_, CO, N_2_, H_2_, NH_3_, C_3_H_8_, C_2_H_2_, CH_2_O, Na^+^, Cl^−^	Global ocean: 250–273 Potential hydrothermal environments: 323 and higher	Ice layer: 30–40 Subsurface ocean: 10	2.8–4.5	10–11	Chondrite core Serpentinisation process Tidal heating	Hydrothermal systems: Lost City (alkaline, serpentinisation driven, 7.5–9 MPa)	Waite et al. (2017), Parkinson et al. (2008), Matson et al. (2007), Porco et al. (2006), Taubner et al. (2015), Hsu et al. (2015) Weber et al. (2023) and Barge and White (2017)
Titan	N_2_, CH_4_, H_2_O, H_2_, Ar, C_2_H_6_, C_2_N_2_, C_6_H_6_, NH_3_	Surface: 95 Subsurface ocean: 235–300	Ice layer: 58–80 Subsurface ocean: 200–300	Ice/water interface: 150 Bottom: 450–800	10–11	Hydrocarbon lakes NH_3_/H_2_O ocean; Core made of silicate rocks; presence of clathrates (methane and ethane); Tidal heating	Caves; Polar sediments; Mariana Trench (274 K, 110 MPa)	Niemann et al. (2005), Fortes (2000), Lopes et al. (2019), Malaska et al. (2022), Norman and Fortes (2010) and Affholder et al. (2025)
Mars	CH_4_, CO_2_, N_2_, Ar, O_2_, CO, H_2_O, ${\mathrm{ClO}}_{4}^{-}$	Surface: 218	Subsurface water: located between 5.4 and 8	Subsurface: 10–303	2–8	Liquid core enriched in Fe and S; Silicate mantle; Soil rich in ${\mathrm{ClO}}_{4}^{-}$	Hypersaline deep mines: Moab Khotsong (3 km deep), Boulty Salt (1.4 km deep)	Sun et al. (2025), Orosei et al. (2018), Taylor (2013), Stähler et al. (2021), Kounaves et al. (2009), Baratoux et al. (2011), Peretyazhko et al. (2018) and Merino et al. (2019)

Subsurface geomicrobiological research has also interrogated caves because of their ubiquity and diversity on the Earth's surface and their occurrence on extraterrestrial bodies (Northup and Lavoie 2001). Caves offer protection from surface stressors such as aridity and radiation, facilitate microbial access to disequilibrium, and tend to concentrate water, carbon and minerals necessary for life (Northup and Lavoie 2001). Caves have been identified across the solar system from our Moon to Mars, and recently Titan, and should be expected wherever dissolution of rocks or solidification of lava occur (Boston et al. 2001; Léveillé and Datta 2010; Malaska et al. 2022). Geomicrobiological research in terrestrial caves has revealed novel metabolic diversity including previously undocumented metabolisms such as sulphur comproportionation (Aronson et al. 2025; Jones and Macalady 2016; Macalady et al. 2007) arising from extreme conditions, widespread evidence of carbon fixation (Frumkin et al. 2023; Selensky et al. 2021), chemolithoautotrophy, utilisation of trace atmospheric gases (Bay et al. 2024), and strong regional selection in patterns of biogeography (Osburn et al. 2023).

Similarly, many aquatic environments serve as analogues to potential habitats on ocean worlds (Glass et al. 2022). Hypersaline ices on oceans and lakes may be analogues for planetary ice‐brine environments (Babin et al. 2025; Buffo et al. 2022). Anoxic marine water columns, such as those of the west coast of Central and South America (Ulloa et al. 2012), may occur kilometres beneath the ice of Europa, Ganymede, Callisto, Titan and Enceladus (Nimmo and Pappalardo 2016). Hydrothermal vents have attracted research interests in extraterrestrial life because they might represent potential analogues for seafloor environments on Europa and Enceladus (Chyba and Phillips 2001; Hsu et al. 2015; Marion et al. 2003; Waite et al. 2017; Weber et al. 2023; Wu et al. 2025) (Table 1). Furthermore, hypotheses about origins of life on Earth and elsewhere posit a central role for hydrothermal environments (Georgieva et al. 2021; Sojo et al. 2016). Vents systems such as those occurring at Lost City field, the Mid‐Cayman Rise and Guaymas Basin feature very diverse chemistries and microbiomes and offer valuable insights into the types of hydrothermal activity that may exist on Europa and Enceladus. Together, these systems represent end‐member examples of hydrothermal environments that can inform models of subsurface ocean activity on icy moons (Table 1).

## 4 Concerted Efforts Are Needed to Investigate Subsurface Environments That Remain Understudied

Subsurface ecosystems are notoriously difficult to access. Terrestrial subsurface research typically focuses on opportunistic access to the subsurface via mines, deep boreholes or dedicated drilling efforts. The International Continental Drilling Programme (ICDP) was founded in 1996, stemming from national efforts which began in the mid‐1980s (Harms and Emmermann 2007). The ICDP follows a workshop model to facilitate proposal writing and evaluation and has supported the development of ~60 drilling projects worldwide (Table S1). While a need to focus on subsurface biology was part of the motivation for forming this international coalition, microbiology focused drilling projects started with the completion of the Mallik 5L‐38 well in Arctic Canada in 2002, followed by the Chesapeake Bay impact structure in 2005, and many more thereafter (Horsfield et al. 2007). In addition to dedicated scientific boreholes, continental subsurface investigation also relies on pre‐existing means of access. Petroleum reservoirs provide insights into sedimentary subsurface ecosystems shaped by long‐term isolation and hydrocarbon‐rich environments (Bonch‐Osmolovskaya et al. 2003; Kaster et al. 2009; L'Haridon et al. 1995; McIntosh et al. 2023). Drinking water wells and monitoring wells provide widespread access to organic carbon poor aquifer systems (González‐Rosales et al. 2025; Ruff et al. 2023; Wang et al. 2025). Further, boreholes drilled into mine tunnels offer valuable opportunities to study deep microbial life under different geological and environmental conditions (Baker et al. 2003; Beckmann et al. 2011; Lollar et al. 2019; Osburn et al. 2019).

**FIGURE 2:**
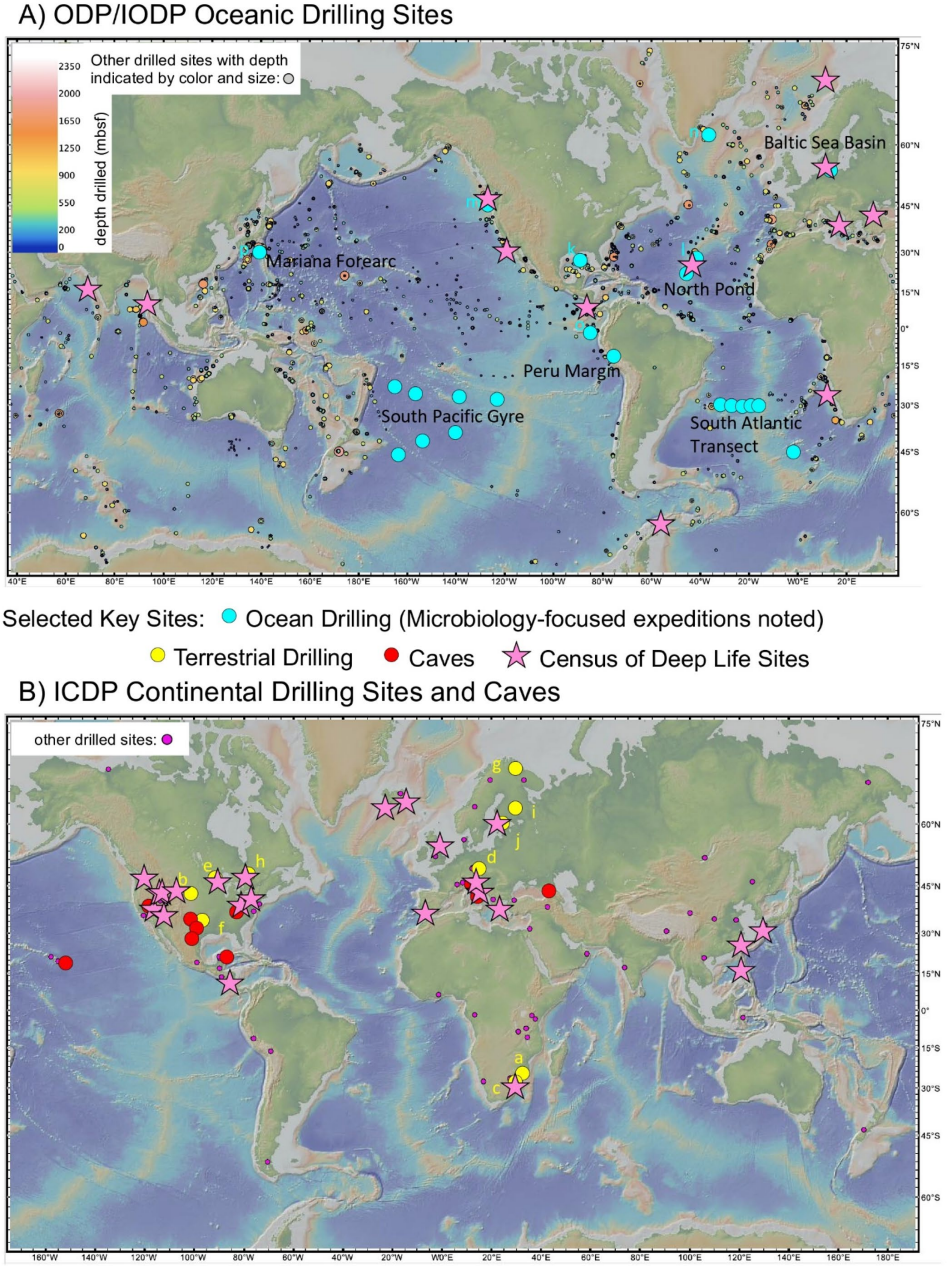
Distribution and locations of subsurface drilling sites. (A) Location of Ocean Drilling Programme (ODP) and International Ocean Drilling Programme/Integrated Ocean Discovery Programme (IODP) drilling sites. Colour and size of location markers both indicate depth drilled in metres below seafloor (mbsf). (B) Location of caves of interest and International Continental Drilling Programme (ICDP) drilling sites, indicated with magenta circle markers. Boreholes, mines and caves of interest (also noted in this figure) are indicated by the larger stars: ocean drilling boreholes in blue, continental drilling boreholes in yellow and caves of interest in red.

Concerning marine subsurface ecosystems, project MoHole (Table S1) achieved the first deep drilling of the ocean floor in 1961, reaching a depth of 183 metres below the seafloor (mbsf) (Greenberg 1964; Lill 1961). The Deep Sea Drilling Project (DSDP) followed in 1966 (Table S1) by conducting extensive drilling and coring across the Atlantic, Indian and Pacific Oceans. Several milestones have been reached, with drilling depths extending up to 1000–2000 mbsf and compelling evidence for microbial life at these depths (Inagaki et al. 2015; Johan Lissenberg et al. 2024; Parkes et al. 2000). Today the sites of subsurface research are globally distributed (Figure 2) and provide access to a broad range of environments, yet there is much potential to discover unseen ecosystems. Additionally, the retirement of flagship drilling vessels such as the D/V JOIDES Resolution has reduced some access to studying subseafloor marine environments (Benningfield 2023), while other programmes (such as IODP3 and new programmes associated with the *D/V Meng Xiang*) are ramping up (Normile 2024). Future endeavours to understand life in the largest habitable environment on Earth (the marine subseafloor sediment and crust), which too are analogues for extraterrestrial environments, will rely on such drilling expeditions.

**FIGURE 3:**
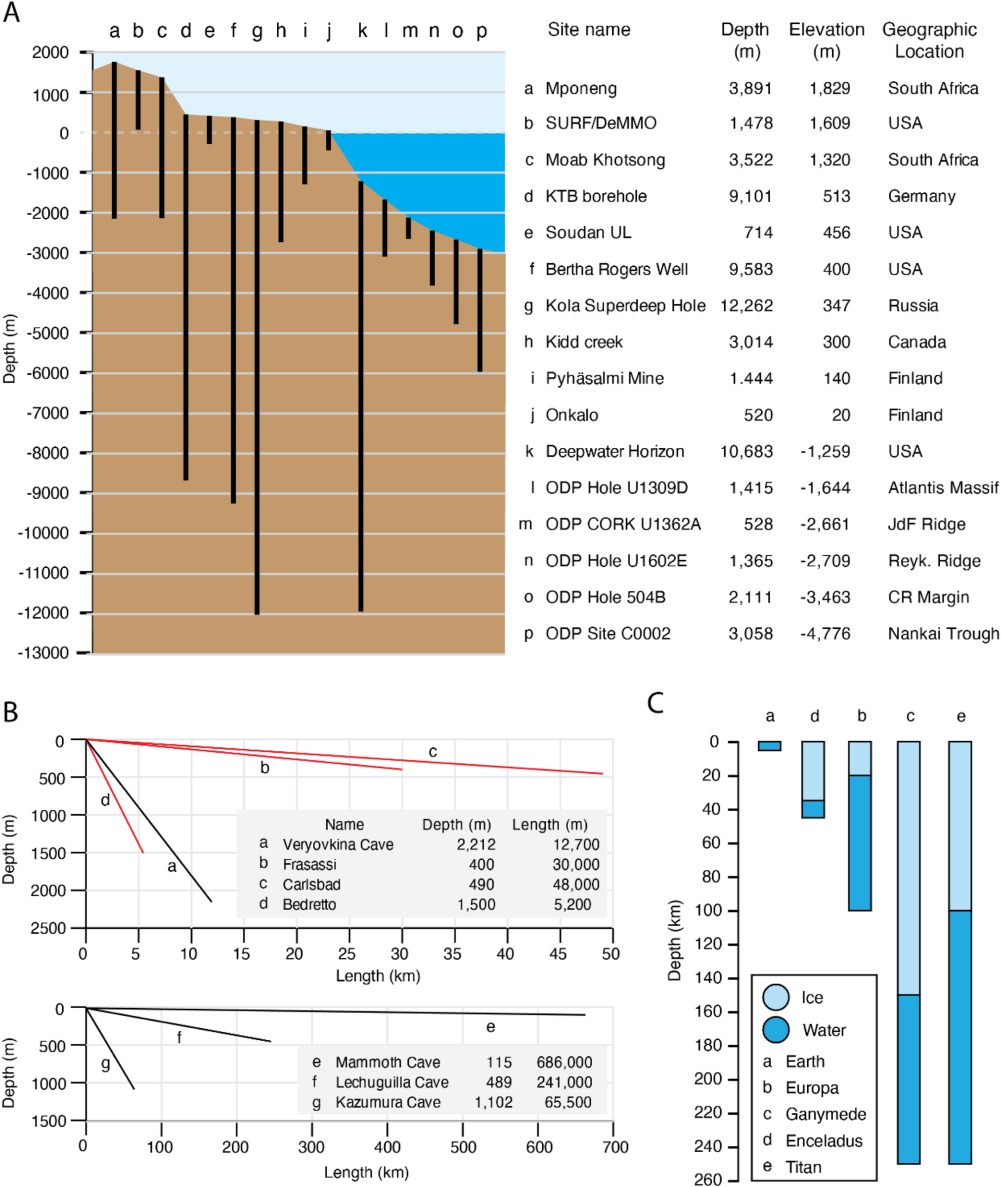
Overview of (A) depth of selected drilled boreholes and mines for comparison, (B) depth and length of selected caves and tunnels. Relatively well studied environments are in red. Note the upper and lower panel have different axes. (C) Thickness of ice sheets and water depths on planetary bodies. Data originates from sources and references given in Table 1.

We have barely penetrated Earth's crust (Figure 3A) and only a minute portion of subsurface rock formations have been sampled. Continued efforts will be required to sample the true diversity of environments present in Earth's subsurface. Observing and characterising subsurface ecosystems in situ has remained a challenge, particularly considering the limitations due to pressure. IODP boreholes and wells that are outfitted with a Circulation Obviation Retrofit Kit (CORK) along with observatories in terrestrial mine and cave systems provide opportunities for long‐term in situ observations (Figure 3A,B). These technologies are windows into the subsurface to study the composition, diversity and processes of subsurface/subseafloor life, and the physical and chemical limits to habitability in these analogue systems. Long‐term in situ observatories at temperature, depth and pressure are necessary to understand dynamic processes which occur in this vast biome, especially as they face some of the same challenges as the technologies we wish to deploy on other worlds. However, long‐term experimental deployments are not yet widely used. Within subsurface environments that have been visited by humans or by drilling technologies (Figure 2), only a small fraction including CORKs allow for repeat visits, but even these require significant time and resource investments with high associated costs. Because of these difficulties, portions of the subsurface remain unworkable for long‐term or repeated sampling. As a result, most current studies are constrained by narrow spatial and temporal coverage, relying on isolated time points. The substantial costs and logistical demands are major factors limiting the deployment of long‐term observatories to subsurface sites. These limitations enlarge sampling biases and limit the reproducibility of investigations, highlighting the need for broader, more systematic deployment of long‐term observatories to accurately assess subsurface habitability and guide interpretations relevant to other planetary bodies.

One possible way to maintain or expand observatories and investigations is to partner with industry and governmental efforts to collect samples either during drilling or after infrastructure has been established. Microbial investigations as part of record‐breaking subsurface endeavours, like the efforts to drill the deepwater horizon borehole (Figure 3), could be used to better understand the spread and diversity of life and habitable environments on Earth. The development of dedicated programmes that connect companies with researchers through calls for expressions of interest or specific workshops should be more widely adopted to strengthen collaborations within academia and between researchers and industry. Subsurface and space research are inherently interdisciplinary—relying on microbiology, geochemistry, engineering and planetary science. Building multi‐institutional networks will be critical to advance technological development, improve sampling strategies and enhance our understanding of both Earth's deep biosphere and the origin of life, while also improving the detection potential for extraterrestrial biosignatures.

Another way of advancing subsurface science is to further identify synergies with research in space exploration. Many of the problems that the two disciplines face are similar, for example low biomass ecosystems and samples, contamination, environmental extremes, instrument communication, autonomous sample collection and remote analysis.

Characterising endemic life in subsurface environments—and potentially in extraterrestrial settings—remains a challenge, particularly due to the risk of contamination (Kieft 2014). In the subsurface, low biomass complicates the detection of indigenous microbial communities. These issues also apply to astrobiological exploration, where the detection of potential biomass and biosignatures in extraterrestrial subsurface environments (e.g., Mars, Europa, Enceladus) faces similar or even more stringent constraints. In both contexts, contamination from surface‐derived or human‐associated microorganisms can obscure indigenous biological signals. For planetary missions, forward contamination by terrestrial microbes is a critical concern, as microorganisms have demonstrated resilience to extreme environmental stressors (Rampelotto 2010) and, in some cases, the capacity to survive and even grow in space conditions such as UV radiation or space vacuum (Deshevaya et al. 2024; Szydlowski et al. 2024). Mitigation strategies developed for subsurface research—such as sterile drilling systems or contamination tracers (Kallmeyer 2017; Labonté et al. 2025)—are increasingly informing planetary protection practices. Similarly, space missions adopt rigorous contamination control measures, including spacecraft sterilisation, cleanroom assembly and microbial monitoring, which may also benefit subsurface investigations on Earth by setting higher standards of cleanliness. Contamination risks do not end at the point of sample collection: samples returned from either terrestrial subsurface environments or space missions remain vulnerable to surface‐ or laboratory‐introduced contamination during recovery, transport and analysis. As such, robust containment strategies and meticulously controlled handling environments are essential for preserving sample integrity. Importantly, practices developed in one domain increasingly inform and inspire those in the other, highlighting the mutual benefit between Earth‐ and space‐based contamination control efforts.

Another notable challenge faced by space and subsurface research alike concerns remote exploration and technology development. Concerted efforts in that regard have led to the development and successful deployment of three exploration robots—designed for space missions—to map and explore caves on Earth (Domínguez et al. 2025). The ROBEX programme (https://www.robex‐allianz.de/en/) is another prominent example in which researchers from diverse fields collaborated to develop autonomous robotic solutions suitable for missions underwater and in space (Dietrich et al. 2014; Sommer et al. 2015). In the future, such collaborations should be expanded to engineer new drilling and sampling technologies. These technological developments must be accompanied by clearly defined research priorities, including (i) preserving samples' integrity through in situ storage (pressure, temperature, etc.); (ii) enhancing robotic autonomy and mobility to navigate confined and difficult‐to‐access environments; (iii) advancing compact, low‐power analytical tools capable of detecting chemical and biological indicators of life in remote settings; and (iv) advancing contamination‐controlled drilling and sampling systems suitable for low‐biomass and remote environments.

To access and sample extraterrestrial subsurface environments, it is necessary to develop and rigorously test autonomous drilling technologies on Earth. The water table, and thus the potential habitable zone, in the rock subsurface of Mars is currently estimated to be located anywhere between 4 and 20 km depth (Butturini et al. 2025; Wright et al. 2024) (Table 1), while the liquid water of the icy moons Europa, Enceladus, Ganymede and Titan is estimated to exist under 20–150 km of ice (Figure 3C; Table 1). For comparison, the deepest boreholes on Earth reach depths of a mere 10–12 km (Figure 3A), approximately half the minimum depth required to penetrate the ice shells of these moons, although within the realm of reaching the Martian water table. The development of autonomous systems capable of penetrating thick ice layers and retrieving samples from subsurface oceans would not only facilitate the search for life beyond Earth but also enhance our ability to investigate and potentially discover new analogous environments on Earth. Long‐term autonomous samplers could advance both planetary science and Earth‐based research by enabling the collection of larger sample volumes and supporting time‐series analyses to study environmental and ecosystems dynamics. These technologies would significantly expand our capacity for remote exploration and deepen our understanding of subsurface environments.

## 5 Studying Slow or Dormant Life Is Key to Understanding Intraterrestrial (And Extraterrestrial) Microbes

Microorganisms that thrive in subsurface environments do not form completely new major branches in the tree of life (Ruff, de Hrabe Angelis, et al. 2024) nor do they appear to rely on fundamentally novel metabolic pathways (González‐Rosales et al. 2025). Instead, they are understudied but phylogenetically related lineages of surface dwellers. One strategy of subsurface microbes to survive geological time scales and extreme conditions is spore formation (Cramm et al. 2019; O'Sullivan et al. 2015; Wörmer et al. 2019). The main distinguishing feature of subsurface life, however, seems to be the ability to outcompete others because of extremely low metabolic energy requirements (Jørgensen 2011; Morono et al. 2011) and low mortality rates (Bradley et al. 2019), resulting in extensively long generation times of up to millennia (Lever et al. 2015; Lloyd 2021; Lloyd and Steen 2025). These ‘aeonophiles’ (Lloyd and Steen 2025) do not merely tolerate extreme energy limitation, but in fact, seem to have developed strategies to survive (and potentially thrive) under these conditions, and to persist over millennial to geological timescales. Dormancy, metabolic slowdown and energy‐efficient maintenance are central characteristics to life in the deep biosphere (Bird et al. 2019; Jørgensen and Marshall 2016). This mode of prolonged dormancy is a survival‐and‐longevity‐strategy carried out in slow motion (Greening et al. 2019; Lloyd 2021): cells may metabolise at rates equivalent to the bare minimum needed for the repair and turnover of biomolecules, extending their lifespan indefinitely without reproduction (Bradley et al. 2019). The lifestyle and adaptations of subsurface microorganisms suggest that dormancy is not a transient state (Cáceres 1997), but a dominant life strategy in low‐energy environments (Bradley 2025; Lloyd 2021) including Earth's frozen ocean (Babin et al. 2025).

Understanding this mode of life requires rethinking life's operational boundaries. In extraterrestrial settings, survival may depend more on persistence than on growth (Bradley 2025; Bradley et al. 2019; Larowe and Amend 2019). Traditional measures—biomass, productivity, replication frequency—may underestimate or even overlook such forms of life. Instead, stable but minimal energy fluxes, even in environments otherwise considered marginal, could sustain longevity‐oriented biospheres (Hoehler 2007).

Importantly, Earth's deep biosphere is not a fringe case. Microbial biomass in Earth's subsurface far exceeds microbial biomass in Earth's terrestrial and marine surface environments (Bar‐On et al. 2018), suggesting that longevity and slow, persistent life may be the dominant mode of existence on our planet, and potentially far beyond. To investigate this, long‐term experimental approaches are needed. Just as long‐term ecological research sites monitor ecosystems over decades, we may need enrichment cultures and microcosms that operate over years to decades, better reflecting microbial timescales or—as others put it—time as a microbial resource (Lloyd 2021; Lloyd and Steen 2025). Such strategies and approaches will be crucial for understanding how ultra‐slow growing and dormant organisms survive on Earth—and how they might endure beyond it.

## 6 Innovative Cultivation Approaches to Unlock Subsurface (And Extraterrestrial) Diversity

Ocean worlds are subject to high hydrostatic pressure (HHP); therefore, if any life form develops there it would be piezotolerant or even piezophilic. Earth's oceans are home to these forms of life, with an average pressure of 38 MPa. However, to date, only 65 microbial species have been isolated from deep environments, with only 14 being strictly piezophilic microorganisms (Table S2). To accurately simulate the conditions of ocean worlds and assess the potential for life, it is essential to use microbial model organisms that are naturally adapted to pressurised environments. Special emphasis should be placed on incorporating HHP as a critical parameter when investigating microbial communities and isolating new strains from the deep subsurface as decompression can lead to bias and loss of information (Garel et al. 2019; Park and Clark 2002; Quéméneur et al. 2019).

**FIGURE 4:**
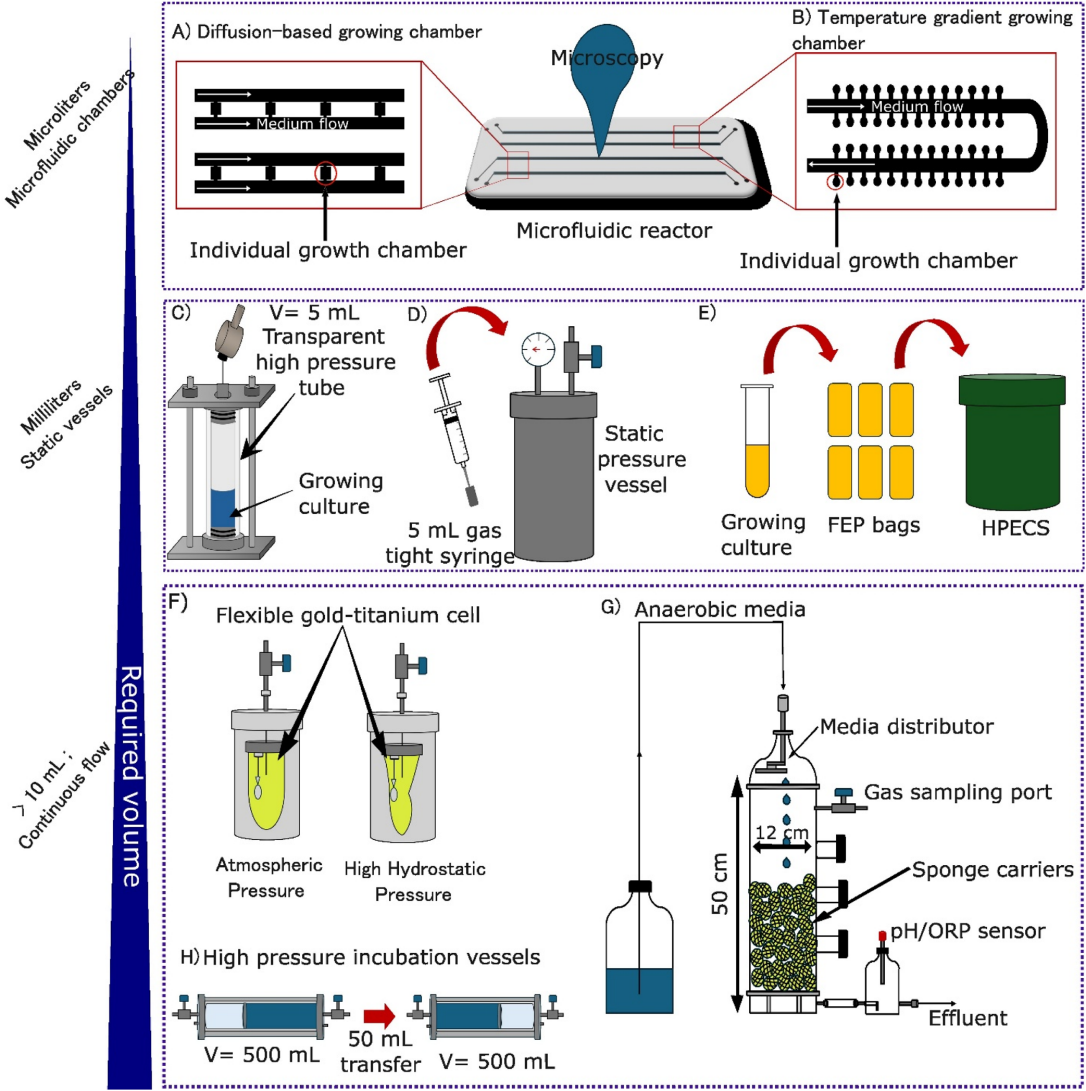
Schematic of selected cultivation tools of interest in subsurface cultivations and astrobiological questions. (A) Microfluidic cultivation chamber (Witting et al. 2024); (B) High‐pressure semi‐transparent microfluidic system for temperature gradient (Cario et al. 2022); (C) Fully transparent sapphire millifluidic cultivation reactor (ICMCB, CNRS, France) (Vidal et al. 2025); (D) Static high‐pressure vessel for cultivation in syringes (Takai et al. 2008); (E) Static High Pressure Experimental Culturing System (HPECS) pressure vessels for Fluorinated Ethylene Propylene (FEP) bags (Malas et al. 2024); (F) Flexible gold‐titanium cell (Zhang et al. 2018); (G) Anaerobic Down‐flow Hanging Sponge (DHS) reactor (Imachi et al. 2019); (H) High‐Pressure Bottles (HPBs) for HHP cultivations (Garel et al. 2019).

Advances in materials science enabled the development of reactors that maintain in situ pressure from sampling through lab‐scale analysis (Garel et al. 2019; Jannasch et al. 1973; Jannasch and Wirsen 1977; Tamburini et al. 2003), eliminating potentially detrimental decompression steps (Figure 4H). Such reactors are critical for studying subsurface microbial diversity (Quéméneur et al. 2019), and for imagining and testing scenarios encountered by life in high‐pressure worlds. Current reactors use different types of materials, accommodate volumes up to hundreds of mL, and withstand pressures up to 158 MPa (Figure 4C–E,H) (Frerichs et al. 2014; Garel et al. 2019; Malas et al. 2024; Sauer et al. 2012; Seyfried et al. 1987; Takai et al. 2008; Tamburini et al. 2003; Vidal et al. 2025; Zhang et al. 2018). These technologies simulate pressure regimes found in Earth's subsurface or potentially in Enceladus or Titan's ice‐ocean interface (Table 1) but fall short of recreating conditions occurring in Europa or Titan's deep oceans. Studying life under these conditions requires tools that reach higher pressures, like gold‐titanium cells (Figure 4F) or Diamond Anvil Cells (DACs) (Oger et al. 2006; Sharma et al., Sharma et al. 2002, Sharma et al. 2009; Zhang et al. 2018). DACs allow in situ observations due to their transparency—a major advantage over traditional tools (Garel et al. 2019; Mori et al. 2023; Takai et al. 2008; Zhang et al. 2018) (Figure 4D,E,H). Advances in transparent HHP set‐ups (Oger et al. 2006; Vidal et al. 2025) (Figure 4C) offer promising solutions for the in situ observation of microbial activity under simulated extraterrestrial conditions and will likely gain widespread attention within the subsurface and astrobiology research community.

Furthermore, microfluidic reactors allow for precise, non‐invasive investigation at small scales (Burmeister et al. 2019; Liu et al. 2012; Morais et al. 2024; Witting et al. 2024) (Figure 4A) and at HHP conditions (Cario et al. 2022) (Figure 4B). Microfluidics approaches allow us to mimic hydrothermal vents, porous sediments or aquifers (Möller et al. 2017; Morais et al. 2016). This type of reactor presents high thermal resistance, with glass or silicium‐borosilicate reactors stable to up to 400°C (Marre et al. 2010). The low volume requirements and relatively simple fabrication make microfluidics tools attractive for the broader scientific community, particularly to researchers interested in subsurface and astrobiological questions.

Continuous‐flow cultivation systems (Figure 4H,–I) are an effective strategy to enrich low‐energy, slow‐growing microorganisms that often defy growth in using conventional batch culture methods. Lab‐based continuous cultivation techniques using low hydraulic retention time replicate the low nutrient conditions encountered in the natural environment by continuously removing metabolic byproducts, selectively enriching for these oligotrophic communities (Imachi et al. 2019). Mimicking subsurface porosity in a large‐scale cultivation system can further support the growth of such organisms (Imachi et al. 2019, 2024), while field deployments of continuous cultivation approaches in mines, enriching subsurface fluids for several months, have proven successful at enriching slow‐growing, mineral‐targeting organisms from fracture fluids (Casar, Kruger, and Osburn 2021; Casar et al. 2020).

Cultivation of endemic subsurface strains under simulated extraterrestrial conditions—such as those of Mars (Harris et al. 2021; Harris and Schuerger 2025) or icy moons (Cosciotti et al. 2019; Malas et al. 2024)—can shed light on the nature and persistence of potential biosignatures. Such studies may also help to predict the types of life forms that could inhabit the deep subsurface of other planetary bodies. Although the development of pressure samplers and novel cultivation reactors has remarkably improved our ability to isolate and study deep subsurface life, several critical limitations persist and must be acknowledged. Studies continue to suffer from sampling biases introduced during sample retrieval, as decompression, temperature shifts and oxygen exposure can selectively influence the composition and activity of native microbial communities (Garel et al. 2019; Miettinen et al. 2015; Mills et al. 2012). These effects potentially result in an underestimation of the native deep biosphere phylogenetic and functional diversity and activities. Culturing challenges further compound this issue: endemic subsurface microorganisms often display extremely slow doubling times, obligate syntrophic interactions and narrow tolerances to changes in pressure or chemistry, usually leading to a selection or enrichment of fast growing or metabolically flexible communities (Schultz et al. 2023). The low biomass of the subsurface adds a further level of complexity by increasing susceptibility to contamination and procedural biases, potentially affecting reproducibility. Furthermore, the use of high‐pressure reactors and cultivation reactors has introduced additional constraints. These devices require specialised materials, fabrication methods and user training, limiting widespread adoption. Differences in pressure limits, thermal tolerance, optical access and sensor integration also influence the accuracy and comparability of results. Meanwhile, microfluidic cultivation devices, while powerful, only allow for manipulation of nano‐ to micro‐litre scale volumes and can be challenging to couple with geochemical monitoring, affecting their quantitative reliability, especially for low biomass settings. Overall, these constraints complicate efforts to extrapolate laboratory findings to other planetary contexts, where the pressure regime, geochemistry and redox state may substantially differ from Earth's analogue environments. A critical evaluation of these methodological limitations is therefore essential when interpreting results and datasets, along with a need to design future experiments to simulate ocean world or rocky planet conditions.

## 7 Life‐Detection and Activity‐Measuring Technologies

Advances in life‐detection technologies are expanding our ability to study organisms that defy cultivation, enabling investigations of in situ processes and metabolisms under natural conditions (Hatzenpichler et al. 2020; Munson‐McGee et al. 2022). Such approaches allow us to examine microbial activities even in communities characterised by extremely long doubling times found in environments where reducing or oxidising power is scarce—conditions that may also characterise extraterrestrial settings.

Technological advances in meta‐omics methods and activity‐based assays have transformed our capacity to characterise complex subsurface communities and to detect active life. A recent breakthrough is the integration of RedoxSensor Green fluorescence measurements with single cell genomic sequencing (Lindsay et al. 2024, 2025; Munson‐McGee et al. 2022). This approach enables the direct measurement of single‐cell respiration rates while simultaneously linking activity with encoded functional potential, providing a powerful means of life detection and characterisation in environments where biomass concentrations are extremely low and conventional genomic methods are often not feasible (Lindsay et al. 2024). Other approaches for detecting microbial activity in subsurface environments include radiotracer incubations (Oremland and Polcin 1982; Whelan et al. 1986), enzyme activity assays (Nunoura et al. 2009), and taxa‐, substrate‐ or cell‐specific activity measurements such as nanoSIMS and BONCAT (Meyer et al. 2024). Collectively, these developments have provided compelling evidence for active microbial communities in subsurface environments, expanding our understanding of the contribution of subsurface life to global biogeochemical cycles and the limits of habitability.

## 8 Concluding Remarks

The search for extraterrestrial life is closely tied to the exploration of Earth's intraterrestrial subsurface environments. Our planet's hidden ecosystems host life forms that thrive under extreme conditions and serve as valuable analogues for astrobiological targets beyond Earth. However, the ultra‐slow and cryptic nature of life in Earth's subsurface challenges traditional microbiological methods, demanding the development of new technologies to access, characterise, and monitor microbial life and its activity. To advance our ability to detect and characterise life elsewhere, we must continue to develop technological innovations, strengthen collaborations between academic institutions and across borders, involve industry partners and space agencies, and implement standardised and bespoke analytical tools for space and subsurface exploration.

## Author Contributions

**Emeline Vidal:** conceptualization, writing – original draft, writing – review and editing, visualization, methodology. **Melody R. Lindsay:** conceptualization, writing – original draft, writing – review and editing, funding acquisition, visualization, methodology. **James A. Bradley:** conceptualization, writing – original draft, writing – review and editing, funding acquisition. **Magdalena R. Osburn:** conceptualization, writing – original draft, writing – review and editing, funding acquisition. **S. Emil Ruff:** writing – original draft, funding acquisition, conceptualization, visualization, writing – review and editing, supervision.

## Funding

This work was supported by a Research Grant from HFSP (RGEC34/2023: https://doi.org/10.52044/HFSP.RGEC342023.pc.gr.168586) to S.E.R. J.A.B. acknowledges support from the Agence Nationale de la Recherche (ANR23‐CPJ1‐0172‐01), the European Research Council (ERC) under the European Union's Horizon Europe Research and Innovation programme (Grant agreement No. 101115755, acronym SIESTA) and a Research Grant from HFSP (RGY0058/2022: https://doi.org/10.52044/HFSP.RGY00582022.pc.gr.153592). M.R.O acknowledges support from the National Science Foundation CAREER (EAR‐2042249) and FRES (EAR‐2120912) programmes, as well as support from her fellowships in the CIFAR Earth 4D: Subsurface Science and Exploration programme and the David and Lucile Packard Foundation. M.R.L. acknowledges support from NASA Exobiology Project 80NSSC23K1355 as well as from post‐expedition award funding from the US Science Support Programme for IODP Expedition 395 (subaward from NSF OCE‐1450528).

## Conflicts of Interest

The authors declare no conflicts of interest.

## Supporting information


**Data S1:** mbt270286‐sup‐0001‐DataS1.docx.

## Data Availability

The authors have nothing to report.
