# Supplementary material for: Subsurface Life on Earth as a Key to Unlock Extraterrestrial Mysteries
Source: Microb Biotechnol. 2025 Dec 18;18(12):e70286. doi: 10.1111/1751-7915.70286 (PMC12712870; doi:10.1111/1751-7915.70286)
Supplement: Supplementary file 1 — Data S1: mbt270286‐sup‐0001‐DataS1.docx. [file MBT2-18-e70286-s001.docx]

Supplementary Table 1 : Summary of international scientific drilling program contributing to understanding Earth’s deep subsurface.

| Program name | Active Years | Objectives | Website | Major Funding agencies |
| --- | --- | --- | --- | --- |
| Project MoHole | 1958 – 1966 | First drilling through the oceanic crust into the Mohorovičić (MoHo) discontinuity to access mantle samples | N/A | National Science Foundation – NSF (USA) |
| Deep Sea Drilling Project – DSDP | 1966 – 1983 | First large scale ocean drilling program to investigate sediment history, plate tectonics and seafloor spreading. | N/A | NSF (USA) |
| Ocean Drilling Program – ODP | 1985 – 2003 | DSDP successor with similar objectives | N/A | NSF (USA) + international partners |
| International Continental Scientific Drilling Program – ICDP | 1996 – present | International program to investigate continental drilling for volcanism, plate tectonics, deep biosphere, hydrology and paleoenvironments | <https://www.icdp-online.org/> | Deutsches GeoForschungsZentrum – GFZ (Germany) as  lead agency + international partners |
| Integrated Ocean Drilling Program – IODP | 2003 – present | International program for ocean drilling addressing Earth system processes, the deep biosphere, crustal development and climate evolution | <https://iodp.org/> | NSF (USA), European Consortium for Ocean Research Drilling (ECORD) and Japan’s Ministry of Education, Culture, Sports, Science and Technology (MEXT) + international partners |
| Center for Dark Energy Biosphere – C-DEBI | 2009-2021 | Program to investigate marine geology, and deep biosphere. | <https://www.darkenergybiosphere.org> | NSF (USA) |
| Deep Carbon Observatory – DCO | 2009 – 2019 | International program investigating carbon cycling, deep carbon reservoirs and the deep biosphere; relied on drilling efforts | <https://deepcarbon.science> | Alfred Sloan Foundation (USA), additional support of NSF, Department of Energy (DOE) + international partners |
| International Center for Deep Life Investigation –  IC-DLI | 2019 – present | International program investigating the diversity, evolution, energy sources, environmental limits and global distribution of deep subsurface life | <https://icdli.sjtu.edu.cn/> | Institutional support through Shanghai Jiao Tong University (SJTU) |

|  |  |  |
| --- | --- | --- |

Supplementary Table 2: Isolated piezophilic strains to date. Obligate piezophiles are highlighted in bold. Modified from (Cario et al., 2019)

| Domain | Order | Strains | Temperature range (opt), °C | Pressure range (opt), MPa | Environment | References |
| --- | --- | --- | --- | --- | --- | --- |
| Bacteria | Actinomycetales | *Dermacoccus abyssi* MT1.1^T^ | 10-37 (28) | 0.1-40 (40) | Mariana trench sediments (10 989 m) | (Pathom-Aree et al., 2006) |
|  | Alteromonadales | ***Colwellia* sp*.*MT41** | **0-10 (8)** | **38-103 (69)** | **Mariana trench (10 476 m)** | (Yayanos et al., 1981) |
|  |  | ***Colwellia hadaliensis* BNL-1^T^** | **2-10 (10)** | **37-102 (90)** | **Puerto Rico Trench (7 410 m)** | (DEMING et al., 1988) |
|  |  | ***Colwellia marinimaniae* MTCD1^T^** | **6-10 (6)** | **80-140 (120)** | **Mariana Trench (10 920 m)** | (Kusube et al., 2017) |
|  |  | ***Colwellia piezophila* Y223G^T^** | **4-10 (10)** | **40-80 (60)** | **Japan trench sediments (6 278 m)** | (Nogi et al., 2004) |
|  |  | *Colwellia piezophila* Y25E^T^ | 10 | 0.1-80 (60) | Japan trench sediments (6 278 m) | (Nogi et al., 2004) |
|  |  | *Moritella* sp*.* strain PE36 | 0-10 (10) | 0.1-70 (41) | Pacific ocean (3 584 m) | (Kato et al., 1995) |
|  |  | *Moritella abyssi* 2693^T^ | 4-14 (10) | 0.1-50 (30) | Atlantic ocean sediments (2 815 m) | (Xu et al., 2003b) |
|  |  | *Moritella japonica* DSK1 | 10-15 (15) | 0.1-70 (50) | Japan trench sediments (6 356 m) | (Kato et al., 1995) |
|  |  | *Moritella profunda* 2674^T^ | 2-12 (6) | 0.1-50 (30) | Atlantic ocean sediments (2 815 m) | (Xu et al., 2003b) |
|  |  | ***Moritella yayanosii* DB21MT-5** | **10** | **50-100 (80)** | **Mariana trench sediments (10 898 m)** | (Kato et al., 1998; Nogi and Kato, 1999) |
|  |  | *Psychromonas* sp*.* strain CNPT3 | 0-15 (12) | 0.1-85 (52) | Pacific ocean, amphipod  (5 800 m) | (Yayanos et al., 1979) |
|  |  | ***Psychromonas hadalis* K41G^T^** | **6-10 (6)** | **30-90 (60)** | **Japan trench sediments (7 542 m)** | (Nogi et al., 2007) |
|  |  | ***Psychromonas kaikoae* JT7304^T^** | **4-15 (10)** | **20-70 (50)** | **Japan trench sediments (7 434 m)** | (Nogi et al., 2002) |
|  |  | *Psychromonas profunda* 2825^T^ | 2-13 (10) | 0.1-50 (25) | Atlantic sediments ocean (2 770 m) | (Xu et al., 2003a) |
|  |  | ***Shewanella benthica* DB127F** | **10** | **50-100 (70)** | **Izu‒Bonin trench sediments (6 499 m)** | (Kato, 1996) |
|  |  | *Shewanella benthica* DB127R | 10 | 0.1-70 (60) | Izu‒Bonin trench sediments (6 499 m) | (Kato, 1996) |
|  |  | ***Shewanella benthica* DB21MT-2** | **10** | **60-100 (70)** | **Mariana trench sediments**  **(10 898 m)** | (Kato et al., 1998) |
|  |  | *Shewanella benthica* DB5501 | 4-15 (15) | 0.1-70 (60) | Suruga bay sediments (2 485 m) | (Kato et al., 1995) |
|  |  | *Shewanelle benthica* DB6101 | 4-15 (10) | 0.1-70 (50) | Ryukyu trench sediments  (5 110 m) | (Kato et al., 1995) |
|  |  | *Shewanella benthica* DB6705 | 4-15 (15) | 0.1-70 (60) | Japan trench sediments (6 356 m) | (Kato et al., 1995) |
|  |  | *Shewanella benthica* DB6909 | 4-15 (15) | 0.1-70 (60) | Japan trench sediments (6 269 m) | (Kato et al., 1995) |
|  |  | *Shewanella benthica* F1A | 2-10 (8) | 0.1-70 (30) | Water column, Atlantic Ocean (4990 m) | (Wirsen et al., 1986) |
|  |  | ***Shewanella benthica* KT99** | **2** | **40-140 (98)** | **Amphipod colonizing Kermadec trench**  **(9 856 m)** | (Lauro et al., 2007) |
|  |  | *Shewanella piezotolerans* WP2 | 0-20 (15-20) | 0.1-50 (15) | West Pacific sediments (1 914 m) | (Xiao et al., 2007) |
|  |  | *Shewanella piezotolerans* WP3 | 0-28 (15-20) | 0.1-50 (20) | West Pacific sediments (1 914 m) | (Xiao et al., 2007) |
|  |  | *Shewanella profunda* LT13a | 4-37 (25-30) | 0.1-50 (10) | Nankai trench sediments (4 790 m) | (Toffin et al., 2004) |
|  |  | *Shewanella violaceae* DSS12 | 8-15 (10) | 0.1-70 (30) | Ryukyu trench sediments (5 110 m) | (Kato et al., 1995) |
|  | Chromatiales | *Thioprofundum lithotrophica* 106 | 30-55 (50) | 0.1-50 (15) | Black smoker, mid-atlantic ridge (3 626 m) | (Takai et al., 2009) |
|  | Desulfovibrionales | *Desulfovibrio hydrothermalis* AM13^T^ | 20-40 (35) | 10 | Hydrothermal vent, East Pacific Ridge  (2 600 m) | (Alazard et al., 2003) |
|  |  | *Pseudodesulfovibrio piezophilus* C1TLV30^T^ | 15-45 (30) | 0.1-40 (10) | Wood fall, Mediterannean Sea (1 693 m) | (Khelaifia et al., 2011) |
|  |  | *Pseudodesulfovibrio produndus* 500-1^T^ | 15-65 (25) | 0.1-40 (10-40) | Japan sea sediments  (518 m below the sea floor) | (Bale et al., 1997) |
|  | Halanaerobiales | *Anoxybacter fermentans* DY22613^T^ | 44-72 (60-62) | 0.1-60 (20) | Hydrothermal sulfurs deposit, East Pacific Ridge (2 891 m) | (Zeng et al., 2015) |
|  | Lactobacillales | *Carnobacterium* sp*.* strain AT12 | 2 | 0.1-60 (20) | Aleoutinnes Trench (2 550 m) | (Lauro et al., 2007) |
|  |  | *Carnobacterium* sp*.* strain AT7 | 20 | 0.1-60 (20) | Central Indian Ridge (2 415-2 460 m) | (Lauro et al., 2007) |
|  | Oceanospirillales | ***Profundimonas piezophila* YC-1** | **4-14 (8)** | **20-70 (50)** | **Water column, Puerto Rico trench**  **(6 000 m)** | (Cao et al., 2014) |
|  |  | *Halomonas titanicae* ANRCS81 | 2-45 (37) | 0.1-55 (35) | Wilhemina Bay (1 350 m) sediment | (Li et al., 2023) |
|  | Rhodobacterales | *Parasedimentitalea marina* W43^T^ | 4-28 (18) | 0.1-50 (0.1) | New Britain Trench (4 000 m) | (Zhang et al., 2022) |
|  |  | *Paroceanicella profunda* D4M1 | 10-45 (37) | 0.1-70 (10) | Mariana trench water column (10 890 m) | (Liu et al., 2020) |
|  |  | *Piezobacter thermophilus* 106 | 30-55 (50) | 0.2-36 (16) | Black smoker, Mid Atlantic Ridge (3 626 m) | (Takai et al., 2009) |
|  |  | ***Piezobacter thermophilus* 108** | **30-55 (50)** | **16-65 (36)** | **Black smoker, Mid Atlantic Ridge**  **(3 626 m)** | (Takai et al., 2009) |
|  |  | ***Rhodobacterales bacterium* PRT1** | **4-12 (10)** | **10-100 (80)** | **Puerto Rico trench (8 350 m)** | (Eloe et al., 2011) |
|  | Thermotogales | *Marinitoga piezophila* KA3^T^ | 45-70 (65) | 0.1-60 | Hydrothermal vent East Pacific Ridge  (2 630 m) | (Alain et al., 2002) |
|  |  | *Pseudothermotoga elfii* | 45-75 (65) | 0.1-40 (20) | Oil-producing well (1 600-1 900 m) | (Roumagnac et al., 2020) |
|  |  | *Thermosipho japonicus* IHB1^T^ | 45-80 (72) | 0.1-60 (20) | Okinawa Through (972 m) | (Takai and Horikoshi, 2000) |
|  | Vibrionales | *Photobacterium profundum* DSJ4 | 4-18 (10) | 0.1-70 (20) | Ryukyu trench sediments  (5 110 m) | (Nogi et al., 1998) |
|  |  | *Photobacterium profundum* SS9 | 3-15 (15) | 0.1-70 (28) | Sulu trench amphipod (2 551 m) | (Allen and Bartlett, 2002; Delong et al., 1997) |
| Archaea | Archaeoglobales | *Archaeoglobus fulgidus* VC-16^T^ | 60-95 (83) | 0.1-60 (20) | Hydrothermal system | (Oliver et al., 2020; Stetter et al., 1987) |
|  | Methanococcales | *Methanocaldococcus jannaschii* JAL-1^T^ | 50-90 (85) | 0.1-75 (75) | Hydrothermal vent, East Pacific (2 610 m) | (Jones et al., 1983) |
|  |  | *Methanococcus thermolithotrophicus* | 30-70 (65) | 0.1-100 (50) | Heated sediments (0.5 m) | (Bernhardt et al., 1988) |
|  | Methanopyrales | *Methanopyrus kandlerii* 116 | 90-122 (105) | 0.1-50 (20) | Water column, Aleutian trench (2500 m) | (Takai et al., 2008) |
|  | Methanosarcinales | *Methanohalophilus profundi* SLHTYRO^T^ | 12-37 (30) | 0.1-50 (35) | Anoxic and hypersaline Tyro basin, Mediterranean Sea (3 350 m) | (L’Haridon et al., 2020) |
|  | Thermococcales | *Palaeococcus ferrophilus* DMJ^T^ | 60-88 (83) | 0.1-60 (30) | Myojin mound of Ogasawara-Bonin arch, Japan (1 338 m) | (Takai et al., 2000) |
|  |  | *Palaeococcus pacificus* DY20341^T^ | 50-90 (80) | 0.1-80 (30) | Hydrothermal vent, East Pacific (2 737 m) | (Zhang et al., 2013) |
|  |  | *Pyrococcus abyssi* GE5 | 67-102 (96) | 0.1-50 (20) | Hydrothermal vent, Fiji basin  (2 000 m) | (Erauso et al., 1993) |
|  |  | *Pyrococcus kukulkanii* NCB100^T^ | 70-112 (105) | 0.1-80 (40-50) | Guaymas basin (1997 m) | (Callac et al., 2016) |
|  |  | ***Pyrococcus yayanosii* CH1^T^** | **80-108 (98)** | **20-120 (52)** | **Hydrothermal vent, Mid Atlantic Ridge** | (Birrien et al., 2011; Zeng et al., 2009) |
|  |  | *Thermococcus aggregans* TY^T^ | 60-95 (75) | 0.1-30 (20) | Guaymas basin (2 000 m) | (Canganella et al., 1998) |
|  |  | *Thermococcus barophilus* MP^T^ | 48-100 (85) | 0.1-80 (40) | Hydrothermal vent, Mid Atlantic Ridge  (3 550 m) | (Marteinsson et al., 1999) |
|  |  | *Thermococcus camini* Iri35c^T^ | 75-80 (90) | 0.1-50 (10-30) | Hydrothermal vent, Mid Atlantic Ridge  (2 300 m) | (Courtine et al., 2021) |
|  |  | *Thermococcus eurythermalis* A501^T^ | 50-102 (85) | 0.1-70 (0.1-30) | Hydrothermal vent, Guaymas basin  (2 000 m) | (Zhao et al., 2015) |
|  |  | *Thermococcus guaymaensis* TYS^T^ | 56-90 (85) | 0.1-50 (20-35) | Guaymas basin (2 000 m) | (Canganella et al., 1998) |
|  |  | *Thermococcus henrietii* EXT12c | 60-95 (80-85) | 0.1-50 (30) | Hydrothermal vent, East Pacific trench  (2 496 m) | (Alain et al., 2021) |
|  |  | *Thermococcus peptonophilus* DSM 10343 | 60-100 (90) | 0.1-60 (45) | Izu‒Bonin Trench (1 380 m) | (González et al., 1995) |
|  |  | *Thermococcus piezophilus* CDGS^T^ | 60-95 (75) | 0.1-135 (50) | Mid-Cayman Rise (4 964 m) | (Dalmasso et al., 2016) |

**References**

Alain, K., Marteinsson, V.T., Miroshnichenko, M.L., Bonch-Osmolovskaya, E.A., Prieur, D., Birrien, J.L., 2002. Marinitoga piezophila sp. nov., a rod-shaped, thermo-piezophilic bacterium isolated under high hydrostatic pressure from a deep-sea hydrothermal vent. Int J Syst Evol Microbiol 52, 1331–1339.

Alain, K., Vince, E., Courtine, D., Maignien, L., Zeng, X., Shao, Z., Jebbar, M., 2021. Thermococcus henrietii sp. Nov., a novel extreme thermophilic and piezophilic sulfur-reducing archaeon isolated from a deep-sea hydrothermal chimney. Int J Syst Evol Microbiol 71.

Alazard, D., Dukan, S., Urios, A., Verhé, F., Bouabida, N., Morel, F., Thomas, P., Garcia, J.L., Ollivier, B., 2003. Desulfovibrio hydrothermalis sp. nov., a novel sulfate-reducing bacterium isolated from hydrothermal vents. Int J Syst Evol Microbiol 53, 173–178.

Allen, E.E., Bartlett, D.H., 2002. Structure and regulation of the omega-3 polyunsaturated fatty acid synthase genes from the deep-sea bacterium Photobacterium profundum strain SS9, Microbiology.

Bale, S.J., Goodman,’, ’ K, Rochelle, P.A., Fry, J.R.J.C., Weightman,’ And, A.J., 1997. Desulfovibrio profundus sp. nov., a Novel Barophilic Sulfate-Reducing Bacterium from Deep Sediment Layers in the Japan Sea, INTERNATIONAL JOURNAL OF SYSTEMATIC BACTERIOLOGY. International Union of Microbiological Societies.

Bernhardt, G., Jaenicke, R., Ludemann, H., Konig, H., 1988. High Pressure Enhances the Growth Rate of the Thermophilic Archaebacterium Methanococcus thermolithotrophicus without Extending Its Temperature Range, APPLIED AND ENVIRONMENTAL MICROBIOLOGY.

Birrien, J.L., Zeng, X., Jebbar, M., Cambon-Bonavita, M.A., Quérellou, J., Oger, P., Bienvenu, N., Xiao, X., Prieur, D., 2011. Pyrococcus yayanosii sp. nov., an obligate piezophilic hyperthermophilic archaeon isolated from a deep-sea hydrothermal vent. Int J Syst Evol Microbiol 61, 2827–2831.

Callac, N., Oger, P., Lesongeur, F., Rattray, J.E., Vannier, P., Michoud, G., Beauverger, M., Gayet, N., Rouxel, O., Jebbar, M., Godfroy, A., 2016. Pyrococcus kukulkanii sp. nov., a hyperthermophilic, piezophilic archaeon isolated from a deep-sea hydrothermal vent. Int J Syst Evol Microbiol 66, 3142–3149.

Canganella, F., Jones, W.J., Gambacorta3, A., Antranikian4, G., 1998. Thermococcus guaymasensis Spm nov. and Thermococcus aggregans sp. nov., two novel thermophilic archaea isolated from the Guaymas Basin hydrothermal vent site, International Journal of Systematic Bacteriology.

Cao, Y., Chastain, R.A., Eloe, E.A., Nogi, Y., Kato, C., Bartletta, D.H., 2014. Novel psychropiezophilic oceanospirillales species profundimonas piezophila gen. nov., sp. nov., isolated from the deep-sea environment of the puerto rico trench. Appl Environ Microbiol 80, 54–60.

Cario, A., Oliver, G.C., Rogers, K.L., 2019. Exploring the Deep Marine Biosphere: Challenges, Innovations, and Opportunities. Front Earth Sci (Lausanne) 7.

Courtine, D., Vince, E., Maignien, L., Philippon, X., Gayet, N., Shao, Z., Alain, K., 2021. Thermococcus camini sp. Nov., a hyperthermophilic and piezophilic archaeon isolated from a deep-sea hydrothermal vent at the mid-atlantic ridge. Int J Syst Evol Microbiol 71.

Dalmasso, C., Oger, P., Selva, G., Courtine, D., L’Haridon, S., Garlaschelli, A., Roussel, E., Miyazaki, J., Reveillaud, J., Jebbar, M., Takai, K., Maignien, L., Alain, K., 2016. Thermococcus piezophilus sp. nov., a novel hyperthermophilic and piezophilic archaeon with a broad pressure range for growth, isolated from a deepest hydrothermal vent at the Mid-Cayman Rise. Syst Appl Microbiol 39, 440–444.

Delong, E.F., Franks, D.G., Yayanos, A.A.A., 1997. Evolutionary Relationships of Cultivated Psychrophilic and Barophilic Deep-Sea Bacteria, APPLIED AND ENVIRONMENTAL MICROBIOLOGY.

Eloe, E.A., Malfatti, F., Gutierrez, J., Hardy, K., Schmidt, W.E., Pogliano, K., Pogliano, J., Azam, F., Bartlett, D.H., 2011. Isolation and characterization of a psychropiezophilic alphaproteobacterium. Appl Environ Microbiol 77, 8145–8153.

Erauso, G., Reysenbach, A.-L., Godfroy, A., Meunier, J.-R., Crump, B., Partensky, F., Baross, J.A., Marteinsson, V., Barbier, G., Pace, N.R., Prieur, D., 1993. Pyrococcus abyssi sp. nov., a new hyperthermophilic archaeon isolated from a deep-sea hydrothermal vent, Arch Microbiol.

gen nov lODY DEMING, C., Somers, L.K., Straube, W.L., Swartz, D.G., Macdonell, M.T., 1988. Isolation of an Obligately Barophilic Bacterium and Description of a New, System. Appl. Microbiol.

González, J.M., Kato, C., Horikoshi, K., 1995. Thermococcus peptonophilus sp. nov., a fast-growing, extremely thermophilic achaebacterium isolated from deep-sea hydrothermal vents. Arch Microbiol 159–164.

Jones, W.J., Leigh, J.A., Mayer, F., Woese, C.R., Wolfe, R.S., 1983. Methanococcus jannaschii sp. nov., an extremely thermophilic methanogen from a submarine hydrothermal vent, Arch Microbiol.

Kato, C., Li, L., Nogi, Y., Nakamura, Y., Tamaoka, J., Horikoshi, K., 1998. Extremely Barophilic Bacteria Isolated from the Mariana Trench, Challenger Deep, at a Depth of 11,000 Meters, APPLIED AND ENVIRONMENTAL MICROBIOLOGY.

Kato, C., Sato, T., Horikoshi, K., 1995. Isolation and properties of barophilic and barotolerant bacteria from deep-sea mud samples.

Khelaifia, S., Fardeau, M.L., Pradel, N., Aussignargues, C., Garel, M., Tamburini, C., Cayol, J.L., Gaudron, S., Gaill, F., Ollivier, B., 2011. Desulfovibrio piezophilus sp. nov., a piezophilic, sulfate-reducing bacterium isolated from wood falls in the Mediterranean Sea. Int J Syst Evol Microbiol 61, 2706–2711.

Kusube, M., Kyaw, T.S., Tanikawa, K., Chastain, R.A., Hardy, K.M., Cameron, J., Bartlett, D.H., 2017. Colwellia marinimaniae sp. nov., a hyperpiezophilic species isolated from an amphipod within the challenger deep, Mariana Trench. Int J Syst Evol Microbiol 67, 824–831.

Lauro, F.M., Chastain, R.A., Blankenship, L.E., Yayanos, A.A., Bartlett, D.H., 2007. The unique 16S rRNA genes of piezophiles reflect both phylogeny and adaptation. Appl Environ Microbiol 73, 838–845.

L’Haridon, S., Haroun, H., Corre, E., Roussel, E., Chalopin, M., Pignet, P., Balière, C., la Cono, V., Jebbar, M., Yakimov, M., Toffin, L., 2020. Methanohalophilus profundi sp. nov., a methylotrophic halophilic piezophilic methanogen isolated from a deep hypersaline anoxic basin. Syst Appl Microbiol 43.

Li, J., Xiao, X., Zhou, M., Zhang, Y., 2023. Strategy for the Adaptation to Stressful Conditions of the Novel Isolated Conditional Piezophilic Strain Halomonas titanicae ANRCS81. Appl Environ Microbiol 89.

Liu, P., Ding, W., Lai, Q., Liu, R., Wei, Y., Wang, L., Xie, Z., Cao, J., Fang, J., 2020. Physiological and genomic features of Paraoceanicella profunda gen. nov., sp. nov., a novel piezophile isolated from deep seawater of the Mariana Trench. Microbiologyopen 9.

Marteinsson, V.T., Birrien, J.-L., Reysenbach, A.-L., Vernet, M., Marie, D., Gamba~orta, A., Me~sner, P., Sleytr4, U.B., Prieur115, D., 1999. Thermococcus barophilus sp. nov., a new barophilic and hyperthermophilic archaeon isolated under high hydrostatic pressure from a deep-sea hydrothermal vent, Journal of Systematic Bacterio/ogy.

Nogi, Y., Hosoya, S., Kato, C., Horikoshi, K., 2004. Colwellia piezophila sp. nov., a novel piezophilic species from deep-sea sediments of the Japan Trench. Int J Syst Evol Microbiol 54, 1627–1631.

Nogi, Y., Hosoya, S., Kato, C., Horikoshi, K., 2007. Psychromonas hadalis sp. nov., a novel piezophilic bacterium isolated from the bottom of the Japan Trench. Int J Syst Evol Microbiol 57, 1360–1364.

Nogi, Y., Kato, C., 1999. Taxonomic studies of extremely barophilic bacteria isolated from the Mariana Trench and description of Moritella yayanosii sp. nov., a new barophilic bacterial isolate. Extremophiles.

Nogi, Y., Kato, C., Horikoshi, K., 2002. Psychromonas kaikoae sp. nov., a novel piezophilic bacterium from the deepest cold-seep sediments in the Japan trench. Int J Syst Evol Microbiol 52, 1527–1532.

Nogi, Y., Masui, N., Kato, C., 1998. Photobacterium profundum sp. nov., a new, moderately barophilic bacterial species isolated from a deep-sea sediment. Extremophiles 1–7.

Oliver, G.C., Cario, A., Rogers, K.L., 2020. Rate and Extent of Growth of a Model Extremophile, Archaeoglobus fulgidus, Under High Hydrostatic Pressures. Front Microbiol 11.

Pathom-Aree, W., Nogi, Y., Sutcliffe, I.C., Ward, A.C., Horikoshi, K., Bull, A.T., Goodfellow, M., 2006. Dermacoccus abyssi sp. nov., a piezotolerant actinomycete isolated from the Mariana Trench. Int J Syst Evol Microbiol 56, 1233–1237.

Roumagnac, M., Pradel, N., Bartoli, M., Garel, M., Jones, A.A., Armougom, F., Fenouil, R., Tamburini, C., Ollivier, B., Summers, Z.M., Dolla, A., 2020. Responses to the Hydrostatic Pressure of Surface and Subsurface Strains of Pseudothermotoga elfii Revealing the Piezophilic Nature of the Strain Originating From an Oil-Producing Well. Front Microbiol 11.

Stetter, K.O., Lauerer, G., Thomm, M., Neuner, A., 1987. Isolation of Extremely Thermophilic Sufate Reducers: Evidence for a Novel Branch of Archaebacteria. Science (1979) 236, 822–824.

Takai, K., Horikoshi, K., 2000. Thermosipho japonicus sp. nov., an extremely thermophilic bacterium isolated from a deep-sea hydrothermal vent in Japan, Extremophiles.

Takai, K., Miyazaki, M., Hirayama, H., Nakagawa, S., Querellou, J., Godfroy, A., 2009. Isolation and physiological characterization of two novel, piezophilic, thermophilic chemolithoautotrophs from a deep-sea hydrothermal vent chimney. Environ Microbiol 11, 1983–1997.

Takai, K., Nakamura, K., Toki, T., Tsunogai, U., Miyazaki, M., Miyazaki, J., Hirayama, H., Nakagawa, S., Nunoura, T., Horikoshi, K., 2008. Cell proliferation at 122°C and isotopically heavy CH 4 production by a hyperthermophilic methanogen under high-pressure cultivation.

Takai, K., Sugai, A., Itoh, T., Horikoshi, K., 2000. Palaeococcus ferrophilus gen. nov., sp. nov., a barophilic, hyperthermophilic archaeon from a deep-sea hydrothermal vent chimney, International Journal of Systematic and Evolutionary Microbiology.

Toffin, L., Bidault, A., Pignet, P., Tindall, B.J., Slobodkin, A., Kato, C., Prieur, D., 2004. Shewanella profunda sp. nov., isolated from deep marine sediment of the Nankai Trough. Int J Syst Evol Microbiol 54, 1943–1949.

Wirsen, C.O., Jannasch, H.W., Wakeham, S.G., Canuel, E.A., 1986. Membrane lipids of a psychrophilic and barophilic deep-sea bacterium. Curr Microbiol 14, 319–322.

Xiao, X., Wang, P., Zeng, X., Bartlett, D.H., Wang, F., 2007. Shewanella psychrophila sp. nov. and Shewanella piezotolerans sp. nov., isolated from west Pacific deep-sea sediment. Int J Syst Evol Microbiol 57, 60–65.

Xu, Y., Kato, C., Liang, Z., Rüger, H.J., De Kegel, D., Glansdorff, N., 2003a. Psychromonas profunda sp. nov., a psychropiezophilic bacterium from deep Atlantic sediments. Int J Syst Evol Microbiol 53, 527–532.

Xu, Y., Nogi, Y., Kato, C., Liang, Z., Rüger, H.J., De Kegel, D., Glansdorff, N., 2003b. Moritella profunda sp. nov. and Moritella abyssi sp. nov., two psychropiezophilic organisms isolated from deep Atlantic sediments. Int J Syst Evol Microbiol 53, 533–538.

Yayanos, A.A., Dietz, A.S., Van Boxtel, R., 1979. Isolation of a Deep-Sea Barophilic Bacterium and Some of Its Growth Characteristics. Science (1979) 205, 808–810.

Yayanos, A.A., Dietz, A.S., Van Boxtel, R., 1981. Obligately barophilic bacterium from the Mariana Trench (deep sea/high pressure/extreme environment/rate of reproduction).

Zeng, X., Birrien, J.L., Fouquet, Y., Cherkashov, G., Jebbar, M., Querellou, J., Oger, P., Cambon-Bonavita, M.A., Xiao, X., Prieur, D., 2009. Pyrococcus CH1, an obligate piezophilic hyperthermophile: Extending the upper pressure-temperature limits for life. ISME Journal 3, 873–876.

Zeng, X., Zhang, Z., Li, X., Zhang, X., Cao, J., Jebbar, M., Alain, K., Shao, Z., 2015. Anoxybacter fermentans gen. Nov., sp. nov., a piezophilic, thermophilic, anaerobic, fermentative bacterium isolated from a deep-sea hydrothermal vent. Int J Syst Evol Microbiol 65, 710–715.

Zhang, Hongge, Fang, J., Zhang, Hongcai, Cao, J., 2022. Complete genome sequence of a psychrotolerant and piezotolerant bacterium Parasedimentitalea marina W43T, isolated from deep sea water of the New Britain trench. Mar Genomics 61.

Zhang, Xiang, Zhang, Xiaobo, Jiang, L., Alain, K., Jebbar, M., Shao, Z., 2013. Palaeococcus pacificus sp. nov., an archaeon from deep-sea hydrothermal sediment xiang zeng. Int J Syst Evol Microbiol 63, 2155–2159.

Zhao, W., Zeng, X., Xiao, X., 2015. Thermococcus eurythermalis sp. nov., a conditional piezophilic, hyperthermophilic archaeon with a wide temperature range for growth, isolated from an oil-immersed chimney in the Guaymas Basin. Int J Syst Evol Microbiol 65, 30–35.
